# 3D morphometric analysis of the epiglottis using CBCT: age and gender differences

**DOI:** 10.1186/s12880-024-01506-y

**Published:** 2024-11-26

**Authors:** Ceren Özeren Keşkek, Emre Aytuğar

**Affiliations:** 1 Department of Oral and Maxillofacial Radiology, Faculty of Dentistry, Izmir Democracy University, Izmir, Türkiye; 2 Department of Oral and Maxillofacial Radiology, Faculty of Dentistry, Izmir Katip Celebi University, Izmir, Türkiye

**Keywords:** Anatomy, Cone-beam computed tomography, Epiglottis, Morphometry

## Abstract

**Background:**

This study aimed to perform a comprehensive morphometric analysis of the epiglottis using cone-beam computed tomography (CBCT) images, including the determination of epiglottis dimensions, the investigation of shape variations, and the assessment of their relationship with gender and age.

**Methods:**

A retrospective analysis was conducted on high-quality CBCT images from 100 patients, obtained using the NewTom 5G system. In CBCT images, epiglottis thicknesses (right, midline, left) and horizontal angle at three levels (suprahyoid, hyoid, infrahyoid) were measured in axial sections, while the length and vertical angle of epiglottis were measured in midsagittal view. Epiglottis shapes were identified through 3D visualization.

**Results:**

The midline epiglottis thicknesses were 4.68 mm at the suprahyoid level, 5.51 mm at the hyoid level, and 6.80 mm at the infrahyoid levels. Epiglottis thicknesses and length were statistically significantly greater in males. Of the 100 patients, 51 had a normal curvature, 41 had a flat epiglottis, and 8 had an omega epiglottis. The omega-shaped epiglottis was significantly longer compared to both the flat and normal curvature types (*p* = 0.011). There was a positive correlation between age and epiglottis thicknesses at the suprahyoid level and horizontal angles at three levels.

**Conclusions:**

This study visualizes epiglottis morphology and uncovers significant morphometric differences. Males exhibit greater epiglottis thickness and length compared to females, while the omega-shaped epiglottis is notably longer than other types. These findings underscore the need for further investigation into the clinical relevance of these morphometric differences, particularly in improving airway management and refining approaches to swallowing function.

## Introduction

The epiglottis is a leaf-like shaped cartilage located posterior to the hyoid bone and tongue, protruding superiorly [1]. It is a component of the larynx that connects to hyoid bone and thyroid cartilage. Its main function is to protect the airway during swallowing and prevent food or liquids from entering the trachea [2]. The epiglottis is an important anatomical structure that must be considered, especially during intubation. During the procedure, epiglottis may fold, edema may occur, and injuries may even cause hematoma. These iatrogenic injuries can lead to difficulty swallowing, sore throat, and hoarseness, negatively affecting the patient’s quality of life [3]. The clinical importance of epiglottis extends beyond this. Acute epiglottitis is a potentially life-threatening disease involving bacterial inflammation of the supraglottic and epiglottic structures, causing airway obstruction. The most common symptoms are odynophagia, dysphagia and voice change. To avoid life-threatening complications of acute epiglottitis, it must be treated quickly and carefully [4]. Literature reports indicate that the anterior-posterior diameter of the epiglottis in patients with acute epiglottitis differs significantly compared to healthy individuals [5]. It is essential to determine the normal value range of epiglottis thickness to distinguish between normal and abnormal epiglottis and to provide reference for other pathologies.

Current literature includes only a limited number of studies focused on the morphology of the epiglottis, assessing key features such as its length [3, 6], thickness [7], and shape [8, 9]. These studies have employed various imaging modalities, including magnetic resonance imaging (MRI) [3], computed tomography (CT) [6, 7], and ultrasonography [10], to assess the morphometry of the epiglottis. While MRI provides the most accurate depiction of soft tissue shapes, it suffers from noise and long scanning times. CT imaging offers multiple slices in different orthogonal planes with minimal thickness, but its main drawback is the radiation dose. Cone-beam computed tomography (CBCT) is ideal option for 3D imaging, enabling effective differentiation between soft tissues and bony structures [11]. It can reduce radiation exposure by one-fifth while maintaining image quality [12]. CBCT is an alternative to CT for examining oropharynx morphology with lower cost and radiation dose, providing images similar to those obtained with low-dose CT protocols [13]. One of the key advantages of CBCT imaging is its capacity to generate a range of 3D visualizations and multi-planar reformats from isotropic volumetric data. Isotropic image data ensures consistent image quality regardless of the projection orientation of the reformatted slices. This significantly aids in studying the complex 3D anatomy of structures and performing linear measurements with high reliability and precision [14]. However, to date, no study has specifically examined the morphometry of the epiglottis using CBCT. This gap highlights the significance of our research, which aims to leverage CBCT technology to provide new insights into the comprehensive morphology of the epiglottis.

This retrospective study aimed to determine the normal thickness of the epiglottis, determine the frequency of epiglottis shapes on 3D images and investigate their relationship with gender and age in the Turkish subpopulation, using CBCT for analysis.

## Materials and methods

In this study, CBCT images taken for various purposes such as diagnosis of pathological formations in the jaws, planning of dental implants or orthodontic surgery, and examination of impacted teeth at the Department of Oral and Maxillofacial Radiology, Faculty of Dentistry, Izmir Katip Celebi University between January 2020 and December 2020 were retrospectively examined. Of the 125 patients examined, 25 were excluded from the study. The inclusion criteria were clear visualization of the epiglottis, artifact-free CBCT images, and patients aged 18 years and older. The exclusion criteria were the history of surgery or trauma in the head and neck region, presence of pathology in the laryngopharyngeal region, sleep apnea, and closed epiglottis. Sample size calculation was conducted using G*Power 3.1.9.7 software. The minimum sample size was 98 with 0.665 effect size, 5% significance level, and 0.90 power [7]. The patients’ ages were grouped as 18–30, 31–50, and ≥ 51 years.

All images were obtained using a CBCT device (NewTom 5G, Quantitative Radiology, Verona, Italy) operating at 110 kVp. The field of view (FOV) of the evaluated images was 15 × 12 cm and the voxel size was 0.200 mm. Reconstruction and evaluation of images were performed using NNT Viewer version 8.0 computer software (NewTom - Quantitative Radiology; Verona, Italy). All CBCT scans in this study were performed with the patient’s mouth closed and in the supine position. All measurements were made by one observer - an oral and maxillofacial radiologist with more than five years of experience. The intraobserver agreement was assessed using the intraclass correlation coefficient (ICC) with measurements made on twenty randomly selected images after 2 weeks.

**Fig. 1:**
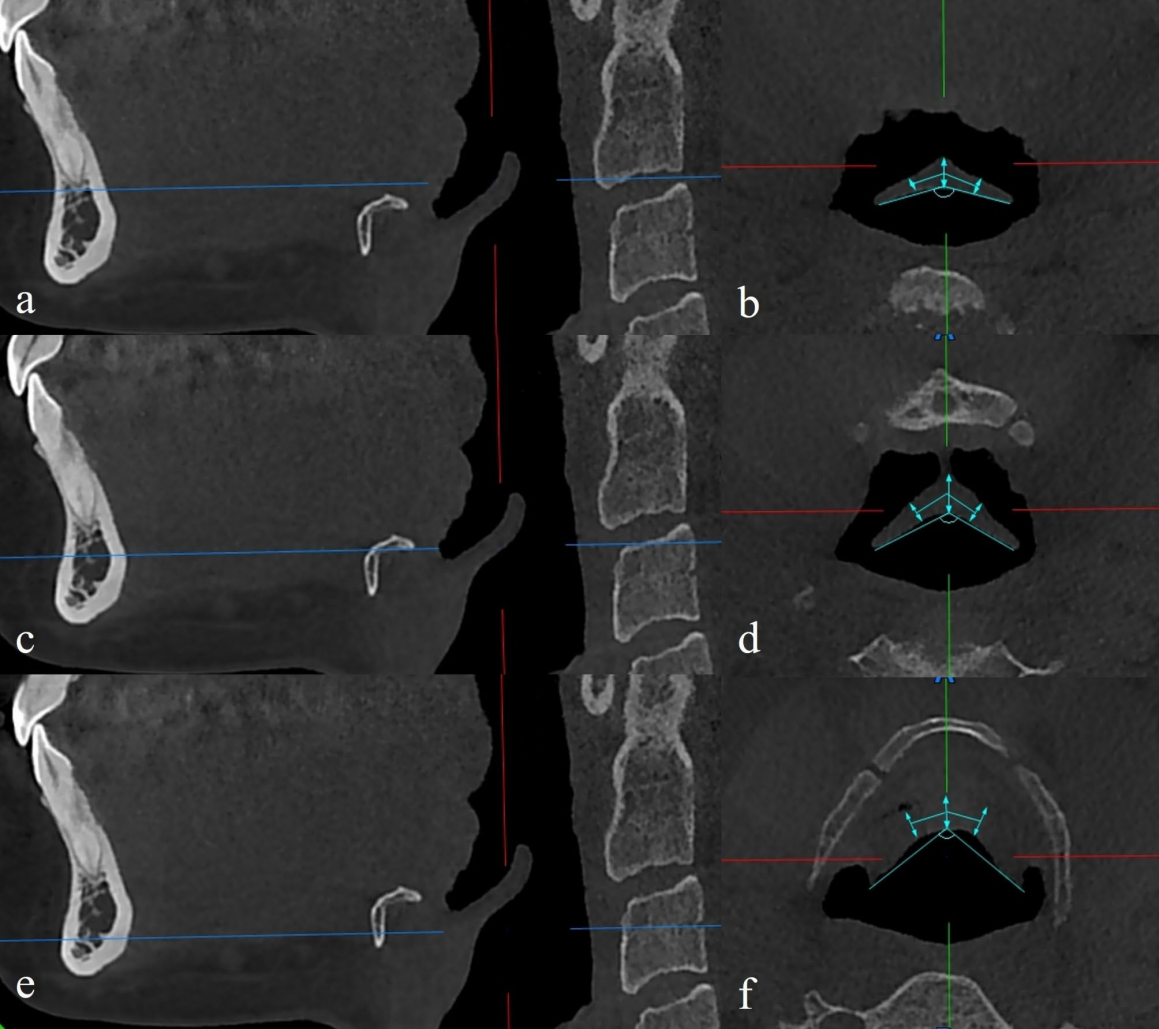
The measurement of epiglottis thicknesses and angles at three levels: (**a**-**b**) Suprahyoid level, (**c**-**d**) hyoid level, (**e**-**f**) infrahyoid level. In sagittal sections, the blue line indicates the level. In axial sections, bi-directional arrows indicate right, midline, and left thicknesses

For image reconstruction, the patient’s position was adjusted parallel to the fourth/fifth cervical disc space. In the sagittal sections, the hyoid level (a line parallel to the upper edge of the hyoid bone), suprahyoid level (4 mm superior to the hyoid level) and infrahyoid level (4 mm inferior to the hyoid level) were determined. Epiglottis thickness was measured in axial sections at these three levels determined in sagittal sections. The epiglottis thickness was determined at 3 points at each level: right side, midline, and left side. The thicknesses on the right and left sides were measured 5 mm away from the midline [7]. The horizontal angle of the epiglottis between the right and left sides was determined (Fig. 1).

**Fig. 2:**
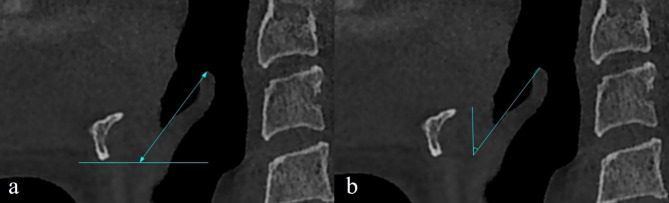
Epiglottis measurements in midsagittal view. (**a**) Epiglottis length, (**b**) epiglottis vertical angle

The length of the epiglottis (from the free edge to the level of the lower border of the hyoid bone) and vertical angle of the epiglottis (the angle between the vertical plane and the long axis extending tangentially from the anterior surface of the epiglottis to the tip of its free edge) were measured in the midsagittal view (Fig. 2).

**Fig. 3:**
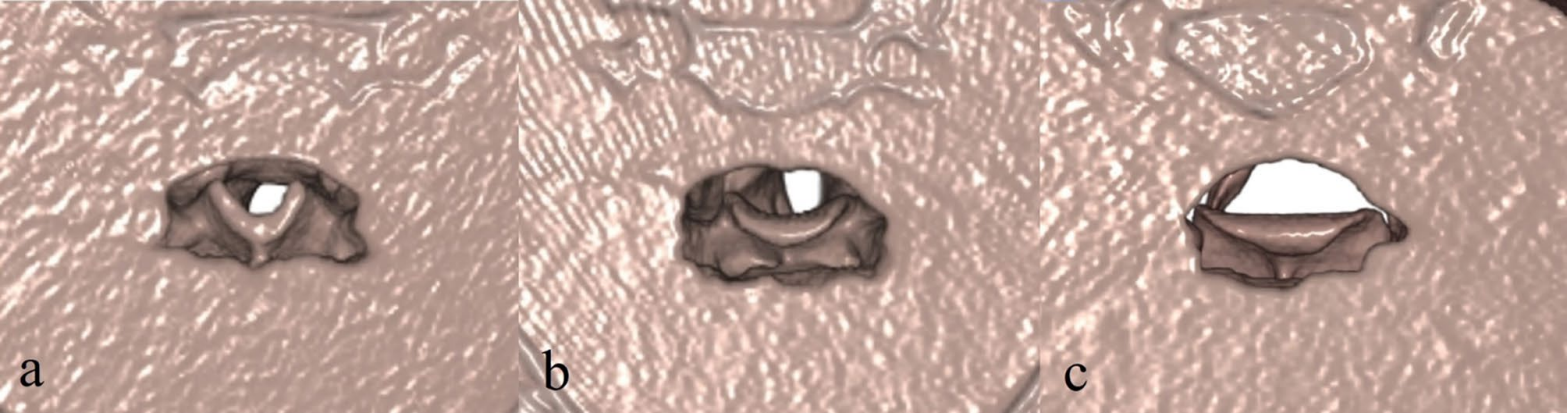
The 3D shapes of the epiglottis. (**a**) Omega, (**b**) normal curvature, (**c**) flat

A 3D reconstruction of the images was performed. The images were examined from a superior perspective and the shape of the epiglottis was noted. Epiglottis shapes were divided into three groups: omega (sharply curved - less than 90°), normally curved and flat epiglottis (Fig. 3). If there was no anterior convexity in the medial part of the epiglottis, it was classified as flat [8].

Statistical analysis was performed with the IBM SPSS Statistics Version 26.0 software package (IBM Corp.; Armonk, New York, USA). The Shapiro-Wilk test indicated that the distribution of the data was normal. Group mean differences were analyzed using t tests and one-way ANOVA, with the Tukey test used for pairwise comparisons. The Chi-square test was used to compare qualitative variables. Correlations between variables were determined using the Pearson correlation coefficient. The significance level in all statistical analyses was *p* < 0.05.

## Results

The intra-observer agreement, assessed by the ICC values (95% CI), ranged from 0.91 to 0.96. In the study, the epiglottis region of 100 patients, 60 women and 40 men, aged between 18 and 85 (mean age: 40.29 ± 17.13 years) was examined.

The comparison of epiglottis measurements according to gender and age groups is shown in Table 1. The epiglottis length and epiglottis thickness at suprahyoid and hyoid levels were statistically significantly higher in men. Conversely, the vertical angle of the epiglottis and the horizontal angle of the epiglottis at the suprahyoid level were greater in women. Significant differences were found in epiglottis thickness at the suprahyoid level and horizontal angles at the three levels according to age groups. There were no significant differences in epiglottis length and vertical angle between age groups. Additionally, epiglottis thicknesses (right, midline, left) increased significantly from the suprahyoid to infrahyoid level (*p* < 0.05).

Among the 100 patients, 51 had a normal epiglottis curvature, 41 had a flat epiglottis, and 8 had an omega-shaped epiglottis. Epiglottis length (*p* = 0.011) and horizontal angles (*p* = 0.000) varied significantly by shape, but no difference was found in the vertical angle (*p* > 0.05). The omega-shaped epiglottis was significantly longer than both the flat and normal curvature types (Table 2).

**Table 1:** The comparison of epiglottis measurements according to gender and age groups

	Gender			Age groups				
	Female Mean (SD) *N*:40	Male Mean (SD) *N*:60	*p*	18–30 Mean (SD) *N*:33	31–50 Mean (SD) *N*:37	51+ Mean (SD) *N*:30	*p*	Total
Suprahyoid level								
Right	2.84 (0.71)	3.45 (0.82)	0.000*	2.82 (0.74)^a^	3.08 (0.88)^ab^	3.37 (0.71)^b^	0.026**	3.08 (0.81)
Midline	4.31 (0.86)	5.24 (0.75)	0.000*	4.40 (0.78)^a^	4.62 (0.82)^ab^	5.06 (1.11)^b^	0.019**	4.68 (0.93)
Left	2.74 (0.72)	3.26 (0.64)	0.000*	2.68 (0.61)^a^	3.03 (0.85)^ab^	3.14 (0.63)^b^	0.028**	2.95 (0.73)
Horizontal Angle	139.53 (21.68)	127.35 (32.68)	0.027*	122.64 (27.95)^a^	142.22 (20.73)^b^	138.55 (29.75)^b^	0.006**	134.66 (27.14)
Hyoid level								
Right	3.62 (0.80)	4.30 (0.77)	0.000*	3.72 (0.88)	3.92 (0.95)	4.04 (0.67)	0.315	3.89 (0.85)
Midline	5.22 (1.21)	5.96 (1.02)	0.002*	5.30 (1.29)	5.44 (1.10)	5.84 (1.15)	0.183	5.51 (1.19)
Left	3.50 (0.85)	4.20 (0.60)	0.000*	3.63 (0.85)	3.63 (0.83)	3.87 (0.83)	0.487	3.78 (0.83)
Horizontal Angle	127.35 (20.58)	117.84 (27.30)	0.065	113.71 (20.73)^a^	129.57 (18.62)^b^	126.94 (29.44)^ab^	0.012**	123.55 (23.84)
Infrahyoid level								
Right	4.08 (0.67)	4.71 (0.82)	0.000*	4.30 (0.94)	4.28 (0.65)	4.43 (0.79)	0.737	4.33 (0.79)
Midline	6.65 (1.13)	7.00 (1.15)	0.145	6.62 (1.35)	6.71 (1.03)	7.10 (1.00)	0.212	6.80 (1.14)
Left	3.96 (4.57)	4.50 (0.71)	0.000*	4.19 (0.87)	4.21 (0.71)	4.21 (0.75)	0.995	4.20 (0.77)
Horizontal Angle	114.14 (21.44)	108.33 (21.20)	0.186	102.16 (18.42)^a^	117.47 (19.01)^b^	115.46 (24.17)^b^	0.005**	111.18 (21.43)
Length	22.09 (3.20)	26.05 (3.91)	0.000*	23.40 (4.15)	23.41 (3.99)	24.29 (3.87)	0.601	23.67 (3.99)
Vertical Angle	29.38 (5.90)	33.02 (6.70)	0.005*	32.44 (7.30)	29.21 (4.51)	31.07 (7.18)	0.109	30.84 (6.45)

*Statistically significant at level *p* < 0.05 (independent samples t test), ** Statistically significant at level *p* < 0.05 (ANOVA test)

The lowercase superscript indicates statistical differences within row (Tukey test post hoc analysis), N: Number of patients, SD: Standard deviation

**Table 2:** The relationship between the angles and length of the epiglottis and its shapes

	Shapes of Epiglottis			
	Omega Mean (SD)	Normal Curvature Mean (SD)	Flat Mean (SD)	*p* value
Length	27.65 (3.61)^a^	23.15 (3.81)^b^	23.54 (3.93)^b^	0.011*
Vertical Angle	34.21 (8.15)	31.33 (6.10)	29.57 (6.37)	0.131
Suprahyoid level Horizontal angle	74.86 (14.14)^a^	128.55 (18.09)^b^	153.92 (15.37)^c^	0.000*
Hyoid level Horizontal angle	79.85 (20.36)^a^	116.59 (15.62)^b^	140.73 (16.81)^c^	0.000*
Infrahyoid level Horizontal angle	87.02 (24.81)^a^	106.11 (18.38)^b^	123.75 (17.21)^c^	0.000*

* Statistically significant at level *p* < 0.05 (ANOVA test)

The lowercase superscript indicates statistical differences within row (Tukey test-post hoc analysis), SD: Standard deviation

There was no significant difference in epiglottis shapes by gender (*p* = 0.102), but a significant difference was found across age groups (*p* = 0.000). In the 18–30 age group, 78.8% had a normal epiglottis curvature. In the 31–50 and 51 + age groups, 56.8% and 63.3% respectively had flat epiglottis. Of the omega-shaped epiglottis cases, six were found in individuals aged 18–30, two in those aged 51 and over, and none in the 31–50 group.

Table 3 shows the correlations of age and epiglottis measurements. A weak positive correlation was observed between age and epiglottis thickness at the suprahyoid level, as well as horizontal angles at three levels. There was no significant correlation between age and the length and vertical angle of epiglottis.

**Table 3:** The correlations between epiglottis measurements and age

	*r* value	*p* value
Suprahyoid level		
Right	0.233	0.02*
Midline	0.297	0.003**
Left	0.259	0.009**
Horizontal Angle	0.217	0.03*
Hyoid level		
Right	0.156	0.122
Midline	0.174	0.083
Left	0.109	0.279
Horizontal Angle	0.206	0.04*
Infrahyoid level		
Right	0.048	0.635
Midline	0.141	0.161
Left	0.013	0.895
Horizontal Angle	0.221	0.027*
Length	0.101	0.315
Vertical Angle	-0.046	0.652

*Correlation is significant at the 0.05 level (two-tailed), **Correlation is significant at the 0.01 level (two-tailed), r: Pearson correlation coefficient

## Discussion

In the present study, we retrospectively analyzed the morphometry of the epiglottis using CBCT in 100 patients from the Turkish subpopulation. Our findings provide detailed radio-morphometric values of the epiglottis. To the best of our knowledge, this is the first study to utilize CBCT for the morphological examination of epiglottis. We believe that this study will assist clinicians in diagnosing epiglottic abnormalities more effectively and enhance awareness of this anatomical region among oral and maxillofacial radiologists.

The dimensions and positions of anatomical structures in the oropharyngeal region, including the hyoid bone, epiglottis, soft palate and tongue, vary depending on gravity and therefore the patient’s position. A notable difference between CBCT and CT is the patient’s position during the scan. CT scans are typically performed in the supine position [15]. CBCT allows for greater flexibility, with patients scanned in sitting, standing, or supine positions, depending on the device used [16]. Sutthiprapaporn et al. [17] reported that the epiglottis moves 6.8 mm caudally when transitioning from a supine to an upright sitting position and 3 mm posteriorly when moving from an upright to a supine position. Recognizing these positional differences is essential to prevent potential misdiagnosis. In the present study, all CBCT scans were performed with the patient in the supine position. The supine position is frequently preferred in clinical settings, particularly during procedures like surgery or general anesthesia. Studying the morphology of the epiglottis in this position offers valuable insights for clinicians working in these environments. Furthermore, previous research has also examined epiglottis in the supine position, validating our methodological approach and enabling easier comparisons with other studies. Nonetheless, we recognize that certain aspects, such as the vertical angle of the epiglottis, may vary depending on the patient’s position. Future studies could explore these variations by comparing CBCT images taken in different positions, providing a more comprehensive understanding of epiglottis morphometry.

Pathologies such as cysts [18], papilloma [19], hemangioma [20], schwannoma [21] and even osteosarcoma [22] can occur in the epiglottis region. CBCT images, frequently used in dentistry for various purposes, may incidentally capture epiglottic pathologies. Thus, early detection and referral of patients can be facilitated. Oral and maxillofacial radiologists must be knowledgeable about the anatomical structures and pathologies that may be incidentally encountered in the imaging field.

Obstructive sleep apnea (OSA) is a multifactorial disorder characterized by recurrent obstructive hypopnea and apnea during sleep. Upper airway obstruction includes the velopharynx, oropharyngeal lateral wall, tongue, and epiglottis (VOTE) classification [6]. Recent studies have highlighted the role of the epiglottis in OSA, either alone or in combination with other pharyngeal structures [8]. However, there is no consensus on its significance in the management and evaluation of OSA, and research is ongoing [23, 24]. Kuo et al. [6] examined factors leading to epiglottic collapse (EC) in 35 OSA patients, finding a significant difference in epiglottic length between the EC group (21.2 ± 3.9 mm) and the non-EC group (15.8 ± 2.9 mm) (*p* < 0.001). They noted the potential link between epiglottis collapse and elongated epiglottis. In this study, the overall mean epiglottis length was 23.67 ± 3.99 mm, with men measuring 26.05 ± 3.91 mm and women 22.09 ± 3.20 mm, showing significant gender differences (*p* = 0.000). These measurements were higher than those in Kuo et al.’s study [6], likely due to differences in measurement methods. Kuo et al. [6] measured from the free edge to the base of the epiglottis in a mid-sagittal view without a defined specific reference point. In this study, epiglottis length was measured from its free edge to the lower border of the hyoid bone, which served as the reference point.

Previous studies have evaluated the shapes of the epiglottis in OSA patients [8, 9]. Gazayerli et al. [9] reported that high body weight in OSA patients could lead to an epiglottis deformity (omega shape), causing chronic collapse of the retroglottal airway during sleep. Delakorda and Ovsenik [8], reported that the epiglottis is flat in patients with obstruction at the base of the tongue or the epiglottis. In their study, 52.5% of individuals with OSA had normal curvature, 37.1% had flat, and 11.4% had omega epiglottis. They also noted that the shape of the epiglottis did not differ in the supine position and the awake sitting position during drug-induced sleep endoscopy. In our study, among the 100 patients without a history of sleep-breathing disorders, 51 had normal curvature, 41 had flat epiglottis, and 8 had omega epiglottis. During the CBCT scan, the patients were awake and in supine position. The similarity of epiglottis shape ratios is noteworthy. Additionally, we investigated the relationship between the shape and length of the epiglottis. The mean length of the omega-shaped epiglottis (27.65 ± 3.61 mm) was statistically significantly higher than the other two shapes. Individuals with omega-shaped epiglottis may be predisposed to OSA due to epiglottic collapse, which may result from the length of the epiglottis rather than its shape. More clinical studies are needed to understand the normal morphology of epiglottis and the relationship between epiglottis shapes, length and OSA.

The epiglottis morphology shows distinct differences between children and adults due to developmental and age-related changes. In children, the epiglottis is softer and more flexible, resulting in considerable shape variation. It often takes on an elongated, tubular form, or occasionally an omega shape, which is also commonly observed in cases of laryngomalacia. Laryngomalacia occurs when the supraglottic structures, including the epiglottis and aryepiglottic folds, collapse during inspiration. This collapse leads to respiratory obstruction and sleep apnea [25]. Although some studies, such as that by Cicekcibasi et al. [26], have investigated the morphometric development of the fetal and pediatric larynx, there is a lack of comprehensive studies specifically focusing on the epiglottis morphology in pediatric populations. Our study, which utilized three-dimensional CBCT imaging, has provided valuable insights into the adult epiglottis morphology. However, further research is necessary to explore epiglottis morphology in children. Such studies would enable a deeper understanding of the development and variation of the epiglottis across different age groups.

In study by Baba et al. [7], the thickness of the epiglottis was assessed in 79 patients using CT, revealing midline thicknesses of 3.8, 4.7, and 6.3 mm at the suprahyoid, hyoid, and infrahyoid levels, respectively. They reported that all epiglottis thicknesses were significantly higher in men. In our study, themidline epiglottis thicknesses were 4.68, 5.51 and 6.80 mm at the same levels. Except for the midline thickness at the infrahyoid level, the right, left and midline thicknesses at other levels were significantly higher in men. Compared with the previous study, the greater epiglottis thickness may be due to sample size, differences in imaging techniques, and ethnic diversity. Baba et al. [7] also observed a significant increase in thickness from the suprahyoid level to the infrahyoid level, which aligns with our findings. The greater epiglottis thickness, length, and vertical angle in men may contribute to a larger airway reduction and a higher prevalence of OSA. However, further morphological studies are needed for clearer conclusions.

In the study conducted by Chau et al. [10], which involved fifty adult volunteers, the normal range of epiglottis thickness was evaluated using ultrasonography, with a mean thickness of 2.36 ± 0.20 mm. This value is thinner compared to the epiglottis thickness observed in our study, likely due to differences in the imaging techniques used. Ultrasonographic evaluation is user-dependent, and standardization of measurements can be challenging. Additionally, Chau et al.‘s study [10] utilized two-dimensional ultrasonographic images to assess the epiglottis, whereas our study employed three-dimensional CBCT images, allowing for a more comprehensive and precise evaluation of epiglottis dimensions.

The current results indicate a significant difference in epiglottis thickness at the suprahyoid level across age groups, which differs from the findings of the previous study [7]. These thicknesses also showed a positive correlation with age. Kano et al. [1], in their investigation of age-related changes in the epiglottis using microscopic and macroscopic measurements, reported a significant increase in epiglottic cartilage thickness with age at three height levels. As the loss of epiglottis guidance function may cause aspiration, its flexibility and elasticity are important in swallowing [1]. The observed increase in thickness with age only at the suprahyoid level in our study requires further microscopic studies.

Based on the findings of this study, the potential impact of epiglottis morphometry on intubation difficulties could be an important clinical outcome. Increased epiglottis thickness and length, particularly in male patients with narrower airways, may make visualization during intubation more challenging, potentially leading to more difficult and possibly traumatic intubation attempts. The longer omega-shaped epiglottis also has clinical significance in this context, as it may alter airway anatomy during intubation and create additional challenges. Future studies could further explore the specific effects of epiglottis thickness and length on airway management, intubation, and swallowing function.

This study has limitations due to its retrospective nature. The presence of sleep-related breathing disorders in individuals was recorded in the patient information management system based on their self-reports and was not medically confirmed. Additionally, the body mass index of the patients is unknown. Another important limitation is that environmental and genetic factors specific to the Turkish subpopulation may influence the prevalence and characteristics of anatomical features, making the results less generalizable to other populations.

## Conclusions

This study provides a comprehensive analysis of epiglottis morphology, revealing significant differences in thickness and length between genders. A positive correlation was observed between age and epiglottis thickness at the suprahyoid level and horizontal angles at three levels. The variations in epiglottis shapes, particularly the longer omega-shaped epiglottis, indicate anatomical factors that may influence airway management and swallowing mechanics. Further research is needed to better understand the clinical significance of epiglottis morphometric differences and their implications for diagnosing and managing related disorders.

## Data Availability

The datasets generated and/or analysed during the current study are available from the corresponding author upon reasonable request.

## Abbreviations

CT: Computed Tomography
CBCT: Cone Beam Computed Tomography
MRI: Magnetic Resonance Imaging
OSA: Obstructive Sleep Apnea
EC: Epiglottic Collapse
